# Defect Detection and Depth Evaluation in Ferromagnetic Steel Using a Custom Magnetic Barkhausen Noise–Hysteresis Sensing System

**DOI:** 10.3390/s26216715

**Published:** 2026-10-23

**Authors:** Xiaochu Pang, Saibo She, Wuliang Yin

**Affiliations:** Department of Electrical and Electronic Engineering, The University of Manchester, Manchester M13 9PL, UK; xiaochu.pang@postgrad.manchester.ac.uk (X.P.);

**Keywords:** magnetic Barkhausen noise, magnetic hysteresis, electromagnetic non-destructive testing, defect depth, sensor design

## Abstract

Reliable inspection of ferromagnetic components requires measurements that relate defect indications to defect severity. This study extends a magnetic hysteresis (*B*–*H*) measurement platform with a dedicated magnetic Barkhausen noise (MBN) channel to examine depth-dependent responses in steel. Finite-element analysis was used to investigate changes in magnetic flux density and magnetic field strength caused by defects. Experiments covered crack-like and strip defects with nominal depths of 0.5–12 mm and 0.5–7 mm, respectively, using two probe sizes. The measured magnetic-field magnitude increased with defect depth, consistent with the simulated trend, whereas the MBN burst amplitude generally decreased. The tabulated peak amplitude, root-mean-square value and zero-field amplitude decreased within both defect series. The cycle-mean amplitude also decreased overall, following a small initial increase relative to the no-defect reference. Peak-field and width features increased with depth in both series, with different magnitudes and rates of change. The combined measurements connect changes in the local magnetic field with changes in the detected Barkhausen response and identify amplitude features as useful indicators for relative defect-depth assessment under the investigated conditions.

## 1. Introduction

Steel is widely used in infrastructure, manufacturing, energy and aerospace applications because of its strength, durability and manufacturability. Surface and subsurface discontinuities, including cracks, grooves and voids, can reduce load-bearing capacity and produce local stress concentrations. Non-destructive testing (NDT) is therefore needed during manufacturing and service to identify defects and assess their severity [1,2].

Available NDT methods exploit optical, acoustic, penetrant and electromagnetic interactions. Visual inspection is rapid but generally restricted to accessible surfaces, whereas ultrasonic testing provides information about internal flaws and requires suitable coupling and inspection geometry. Magnetic-particle and penetrant testing provide indications of surface-breaking defects. Recent developments in magnetic-particle testing have used three-dimensional profilometry and digital-model-assisted denoising to improve automatic defect identification [3,4].

Electromagnetic methods are particularly relevant to conductive or ferromagnetic components. Eddy-current studies have addressed lift-off and tilt sensitivity, simultaneous thickness and defect-depth measurement, and depth evaluation using sensor arrays [5,6,7]. Alternating-current field measurement has been developed for crack-angle monitoring and crack quantification under lift-off variations [8,9]. Magnetic-flux-leakage methods have also been used to reconstruct defect opening profiles and inspect defects with different orientations and widths [10,11]. These approaches form part of a broader set of electromagnetic NDT techniques, including metal magnetic memory and magnetic Barkhausen noise (MBN) [12,13].

MBN originates primarily from discontinuous domain-wall motion during magnetisation. Its sensitivity to the local magnetic state makes it useful for examining both geometrical discontinuities and material changes. Vengrinovich et al. investigated the application of the magnetic Barkhausen effect to the evaluation of stresses and structure in ferromagnetic materials [14]. Mandal et al. combined MBN and magnetic-flux-leakage measurements to investigate stress distributions around a racetrack-shaped defect in steel [1]. Other studies have related Barkhausen activity to microstructural defects and elastic or plastic deformation [15,16]. These findings establish the physical sensitivity of MBN, while also motivating direct measurement of the magnetic excitation when relating its response to defect depth.

The question addressed here is how defect depth changes the MBN response when the local magnetic field is measured under the same excitation. A custom sensing system combines MBN acquisition with an existing *B*–*H* measurement architecture. Finite-element analysis characterises the defect-induced magnetic-circuit response, and experiments examine amplitude, peak-field and width features for crack-like and strip defects. This combination provides a physical context for interpreting feature–depth relationships and comparing their behaviour within each defect series.

The following sections present the theoretical background, finite-element model and sensing system, followed by the experimental responses, feature analysis and implications for defect-depth assessment.

## 2. Theoretical Background

### 2.1. Magnetic Hysteresis Model

Ferromagnetic hysteresis reflects the dependence of magnetisation on both the instantaneous magnetic field and the preceding magnetisation history. The Jiles–Atherton (J–A) model describes this behaviour through reversible domain-wall deformation and irreversible displacement against pinning sites [17]. For an isotropic material, the anhysteretic magnetisation provides a reference state towards which the magnetisation evolves. The effective field and the corresponding anhysteretic magnetisation are expressed as:

(1) $$H_{e}=H+\alpha M$$

(2) $$M_{\mathrm{an}}=M_{s}\left[\coth\;\left(\frac{H_{e}}{a}\right)-\frac{a}{H_{e}}\right]$$

where *H* is the internal magnetic field, $H_{e}$ is the effective field, *M* is the total magnetisation, $M_{\mathrm{an}}$ denotes the anhysteretic magnetisation, and $M_{s}$ is the saturation magnetisation. The parameter *a* determines the field scale of the anhysteretic response, while α represents interdomain coupling. The internal field in this model is distinguished from the local surface field measured by the Hall sensor in the experiments.

Following the separated-component formulation of the J–A model [18,19], the total magnetisation is written as:

(3) $$M=M_{\mathrm{irr}}+M_{\mathrm{rev}}$$

(4) $$M_{\mathrm{rev}}=c\left(M_{\mathrm{an}}-M_{\mathrm{irr}}\right)$$

where $M_{\mathrm{irr}}$ and $M_{\mathrm{rev}}$ are the irreversible and reversible components, respectively, and *c* controls the reversible contribution. The field-dependent evolution is described by:

(5) $$\frac{dM_{\mathrm{irr}}}{dH}=\frac{M_{\mathrm{an}}-M_{\mathrm{irr}}}{k\delta -\alpha \left(M_{\mathrm{an}}-M_{\mathrm{irr}}\right)}$$

(6) $$\frac{dM}{dH}=\left(1-c\right)\frac{dM_{\mathrm{irr}}}{dH}+c\frac{dM_{\mathrm{an}}}{dH}$$

where *k* characterises the resistance to irreversible magnetisation changes associated with domain-wall pinning. The direction parameter is δ=+1 for an increasing field and δ=−1 for a decreasing field.

### 2.2. Magnetic Barkhausen Noise (MBN) Theory

MBN arises primarily from abrupt domain-wall displacements as the changing magnetic field drives walls past pinning sites. Unlike the smooth macroscopic hysteresis response, these discontinuous events produce fluctuating magnetic flux that can be detected by an inductive coil. Their occurrence and magnitude depend on the local magnetic state and the distribution of microstructural and stress-related barriers [19,20,21].

The connection between irreversible magnetisation and Barkhausen activity can be expressed through the jump-sum magnetisation $M_{\mathrm{JS}}$, which represents the accumulated magnetisation changes associated with Barkhausen events:

(7) $$\frac{dM_{\mathrm{JS}}}{dt}=\gamma \frac{dM_{\mathrm{irr}}}{dH}\frac{dH}{dt}$$

(8) $$\gamma =\frac{d}{dM_{\mathrm{irr}}}\left[N_{b}\langle \Delta M_{b}\rangle \right]$$

where $N_{b}$ denotes the number of events over the considered interval and $\langle \Delta M_{b}\rangle$ is their mean magnetisation increment. The dimensionless factor γ accounts for the contribution of discontinuous events to the irreversible magnetisation change and incorporates their statistical variability. Equations (7) and (8) link Barkhausen activity to the irreversible differential susceptibility and the magnetic field sweep rate. Consequently, changes in the excitation waveform or frequency can influence the measured signal even when the material condition remains unchanged.

The Barkhausen-related flux induces a pickup coil voltage according to Faraday’s law:

(9) $$u_{\mathrm{BN}}\left(t\right)=-N_{p}\frac{d\Phi_{\mathrm{BN}}}{dt}\approx -\mu_{0}N_{p}A_{\mathrm{eff}}\frac{dM_{\mathrm{JS}}}{dt}$$

where $N_{p}$ is the number of turns in the pickup coil and $\Phi_{\mathrm{BN}}$ is the Barkhausen-related flux per turn. Here, $\mu_{0}$ is the permeability of free space. The approximate expression uses an effective coupling area $A_{\mathrm{eff}}$ to represent the conversion from magnetisation changes to linked flux under fixed coupling conditions. The experimentally recorded signal also depends on electromagnetic attenuation, amplification and filtering. The dedicated MBN channel separates the high-frequency response from the low-frequency magnetisation background by band-pass filtering.

These relationships motivate the combined analysis of hysteresis and MBN measurements. A geometrical defect can alter the local field distribution and flux coupling, thereby modifying the detected Barkhausen response even when the bulk material composition remains unchanged. Amplitude, peak-position and width features can consequently provide complementary indicators of the defect-dependent magnetic response.

## 3. Materials and Methods

### 3.1. Finite-Element Model

**Figure 1:**
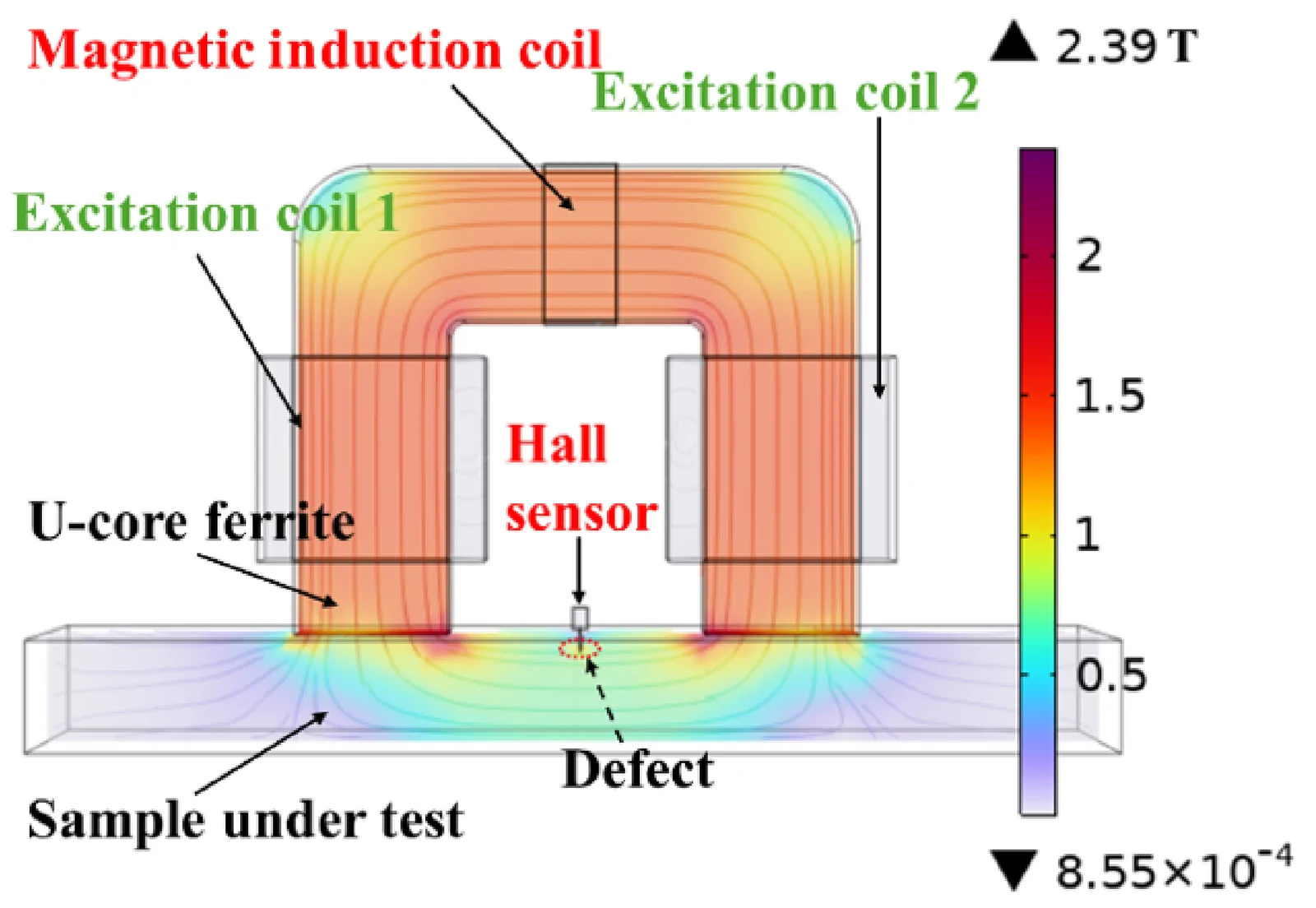
Finite-element model of the magnetic Barkhausen noise (MBN) probe assembly and magnetic flux-density distribution. The region labelled “Hall sensor” represents the idealised field-sampling domain. The colour scale is in tesla.

**Figure 2:**
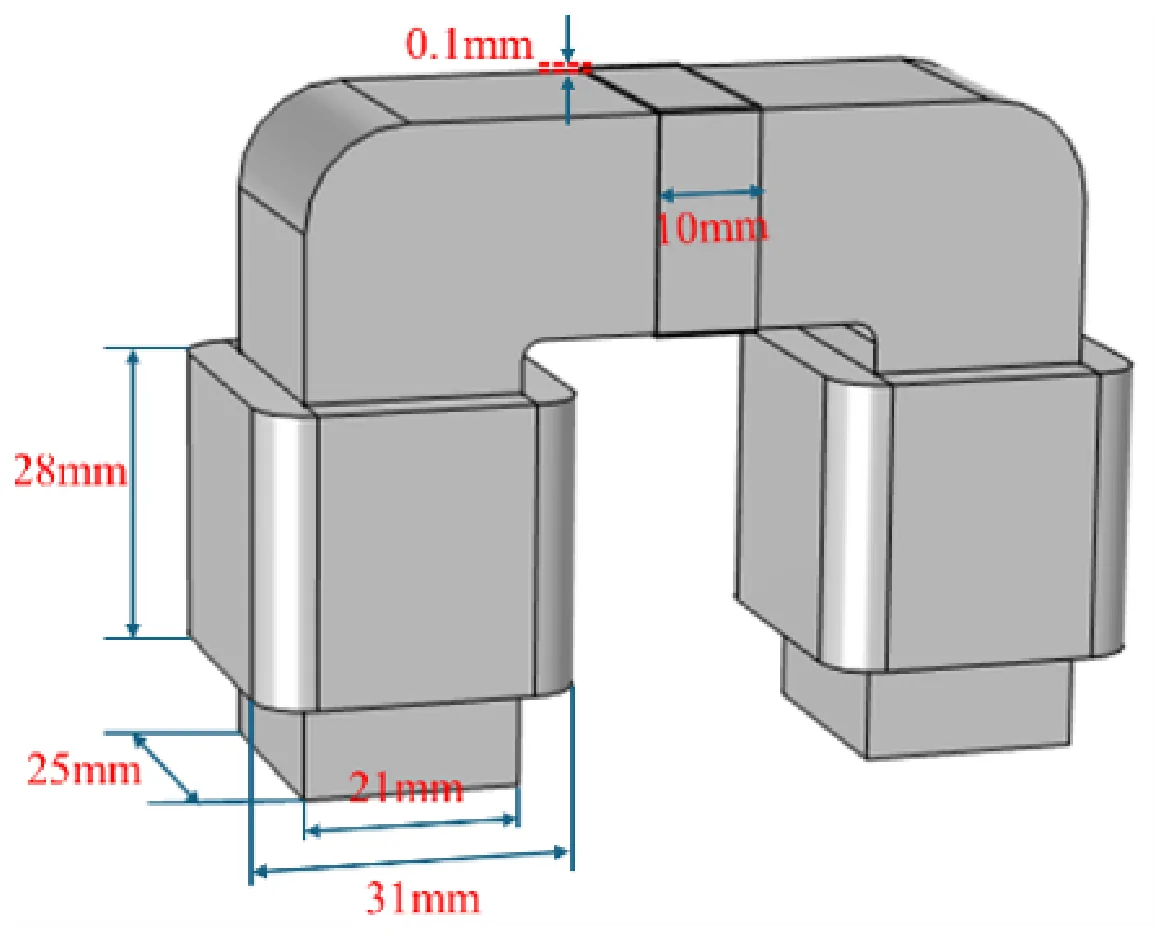
Principal dimensions of the simulated probe assembly.

A three-dimensional probe model was constructed in COMSOL Multiphysics 6.1 using the Magnetic Fields interface of the AC/DC Module. Figure 1 and Figure 2 show the geometry and principal probe dimensions. Two oppositely wound excitation coils were placed on the arms of a ferrite U-core. A magnetic induction coil was positioned at the centre of the yoke, and a Hall-sensor domain was located between the pole faces at the specimen surface. Each excitation coil had 200 turns, a wire diameter of 0.4 mm and a height of 28 mm. The magnetic induction coil had 50 turns, a wire diameter of 0.1 mm and a height of 10 mm, consistent with Figure 2. The magnetic induction coil was constrained to zero current for induced-voltage evaluation. Magnetic flux density *B* and magnetic field strength *H* were used to characterise the macroscopic magnetic-circuit response. Table 1 lists the model parameters. Defect depth was varied while the remaining geometry and excitation were held constant.

The Hall sensor was represented by an idealised field-sampling domain measuring $2\times 2\times 3\;{\mathrm{mm}}^{3}$, located between the U-core pole faces at the specimen surface. This domain was assigned air properties with a relative permeability of $\mu_{r}=1$, as listed in Table 1. It was used to evaluate the local magnetic field at the sensor position and its variation with defect depth. The semiconductor structure, bias current, Hall-voltage generation and signal-conditioning circuitry were not explicitly modelled. Consequently, the simulated response represents the local magnetic field within the sampling domain rather than a directly calculated Hall-sensor output voltage.

**Figure 3:**
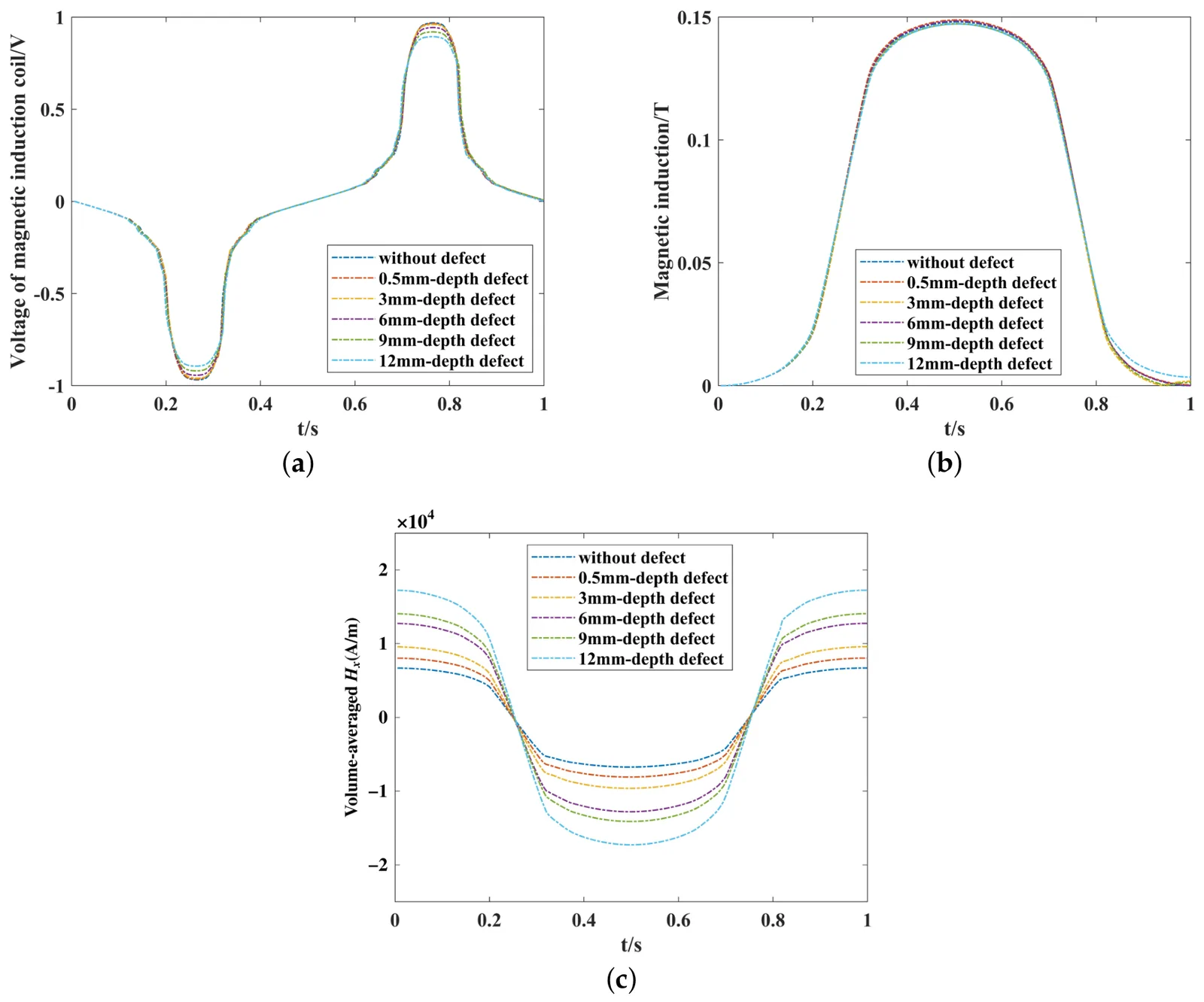
Simulated responses for the no-defect reference and increasing defect depths: (**a**) magnetic induction coil voltage, (**b**) magnetic flux density obtained by integrating the coil response, and (**c**) volume-averaged *y*-component of the magnetic field strength in the Hall-sensor sampling domain. The signed component ${\bar{H}}_{y}$ is reported in A/m.

The field quantity evaluated in the Hall-sensor sampling domain was the signed *y*-component of the magnetic field strength, $H_{y}$, rather than the vector magnitude |H|. The value reported in Figure 3c was the volume-averaged component, defined as

(10) $$\bar{H_{y}}\left(t\right)=\frac{1}{V_{s}}\int_{V_{s}}H_{y}\left(r,t\right)\;dV,$$

where $V_{s}$ is the Hall-sensor sampling volume. The resulting quantity has units of A/m. Positive and negative values indicate opposite field directions along the model *y*-axis. Although a Hall sensor responds to magnetic flux density, the corresponding field components in the air sampling domain satisfy $B_{y}=\mu_{0}H_{y}$ because $\mu_{r}=1$. Therefore, $H_{y}$ was used to characterise the local magnetic excitation at the Hall-sensor position.

The specimen was represented by a linear isotropic material with relative permeability 100 and electrical conductivity $2\times 10^{6}\;S\;m^{-1}$. The U-core was assigned a nonlinear *B*–*H* relation spanning flux densities from 0 to 2.4T and field strengths from 0 to $3.475\times 10^{5}\;A\;m^{-1}$. Magnetic insulation was applied to the exterior boundary of the surrounding air domain. The model used free tetrahedral elements and a boundary-layer mesh on selected specimen faces. The global minimum and maximum element sizes were 5 and 30 mm, respectively. Local refinement used limits of 1 and 20 mm around the probe domains, while the specimen-domain limits were 6.48 and 36 mm. A coil-geometry analysis preceded the time-dependent solution. The transient problem was solved with a fully coupled nonlinear scheme (maximum 15 iterations), a BiCGStab iterative linear solver and a multigrid preconditioner. This model evaluates macroscopic field redistribution; it does not resolve individual Barkhausen events or predict the MBN voltage directly.

Figure 3 compares the simulated responses for the no-defect reference and increasing defect depths. The magnetic induction coil voltage and the corresponding integrated flux-density response decrease overall, whereas the amplitude of ${\bar{H}}_{y}$ in the Hall-sensor sampling domain increases. A deeper discontinuity increases the reluctance of the affected magnetic path and redistributes flux around the defect. This can reduce the flux linked to the magnetic induction coil while increasing the local field amplitude at the Hall-sensor position. The comparison with experiment is qualitative and concerns the direction of change in the magnetic-field amplitude, rather than agreement in absolute magnitude.

### 3.2. Custom MBN–*B*–*H* Sensing System

**Figure 4:**
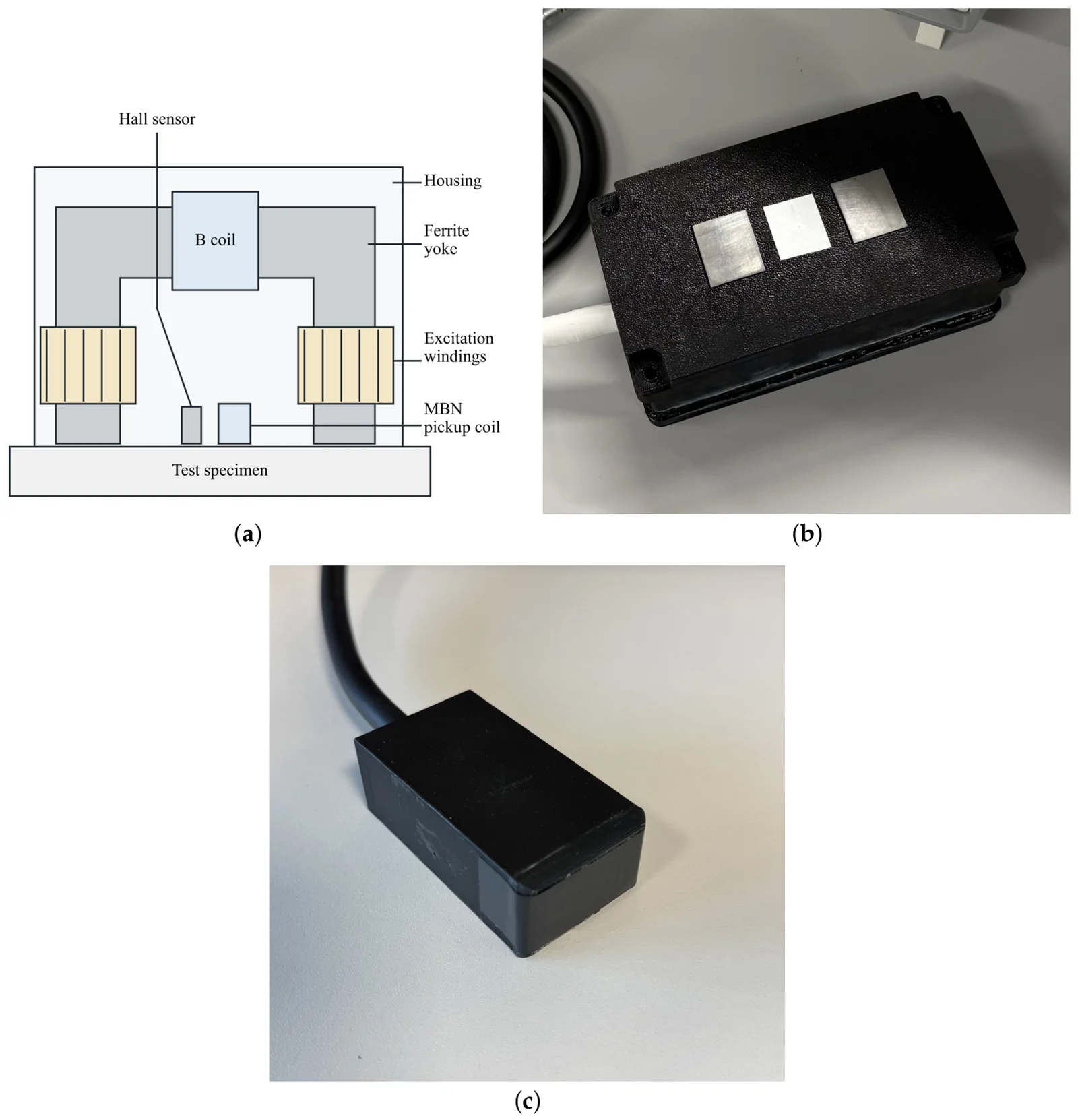
Probe configuration and fabricated sensors: (**a**) schematic of the sensing head, (**b**) large probe, (**c**) compact probe.

The instrument extends the excitation and *B*–*H* acquisition architecture described by Pang et al. [22] with a dedicated MBN channel. Figure 4 shows the sensing-head schematic and the two fabricated probes. A ferrite U-core and excitation windings magnetise the specimen, while a *B* coil, a Hall sensor and an MBN pickup coil provide complementary measurements under a common excitation. The Hall sensor and MBN pickup coil are located between the magnetic poles. Table 2 lists the MBN pickup-coil parameters.

**Figure 5:**
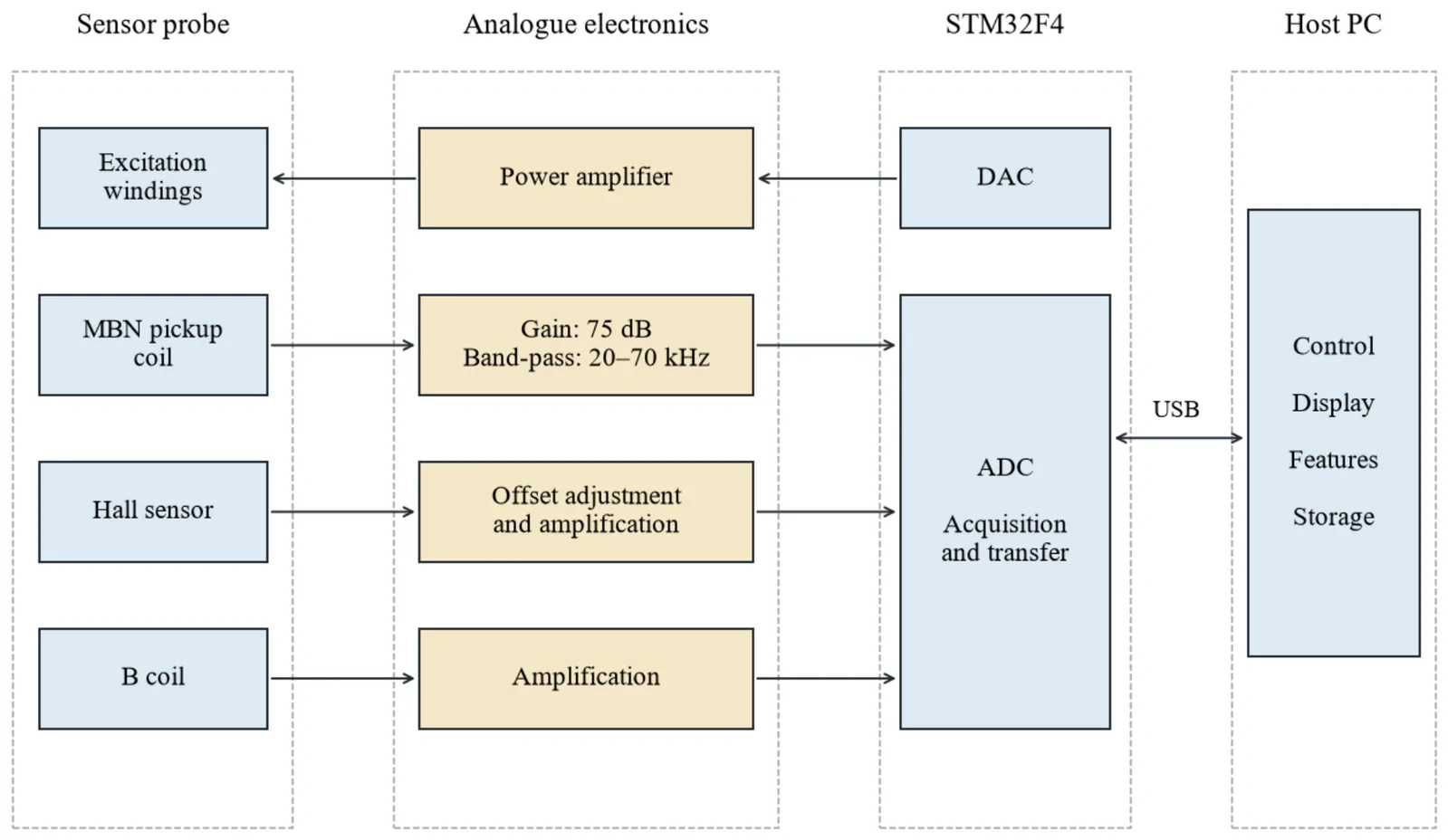
Hardware architecture of the MBN–*B*–*H* measurement system.

As summarised in Figure 5, an STM32F4 microcontroller generates the excitation waveform through a digital-to-analogue converter and power amplifier. The MBN channel provides 75 dB amplification and a 20–70 kHz band-pass filter. The *B*-coil and Hall-sensor outputs are conditioned separately, and the acquired signals are transferred to a host computer for display, processing and storage.

The large probe was used for the plate specimen containing crack-like defects, while the compact probe was used for the smaller specimen containing strip defects. The compact probe has overall dimensions of 70×35×25mm and a magnetic excitation region of 28×15mm. Its MBN pickup coil has a nominal effective sensing region corresponding to a circle of 2 mm diameter.

### 3.3. Experimental Setup and Specimens

**Figure 6:**
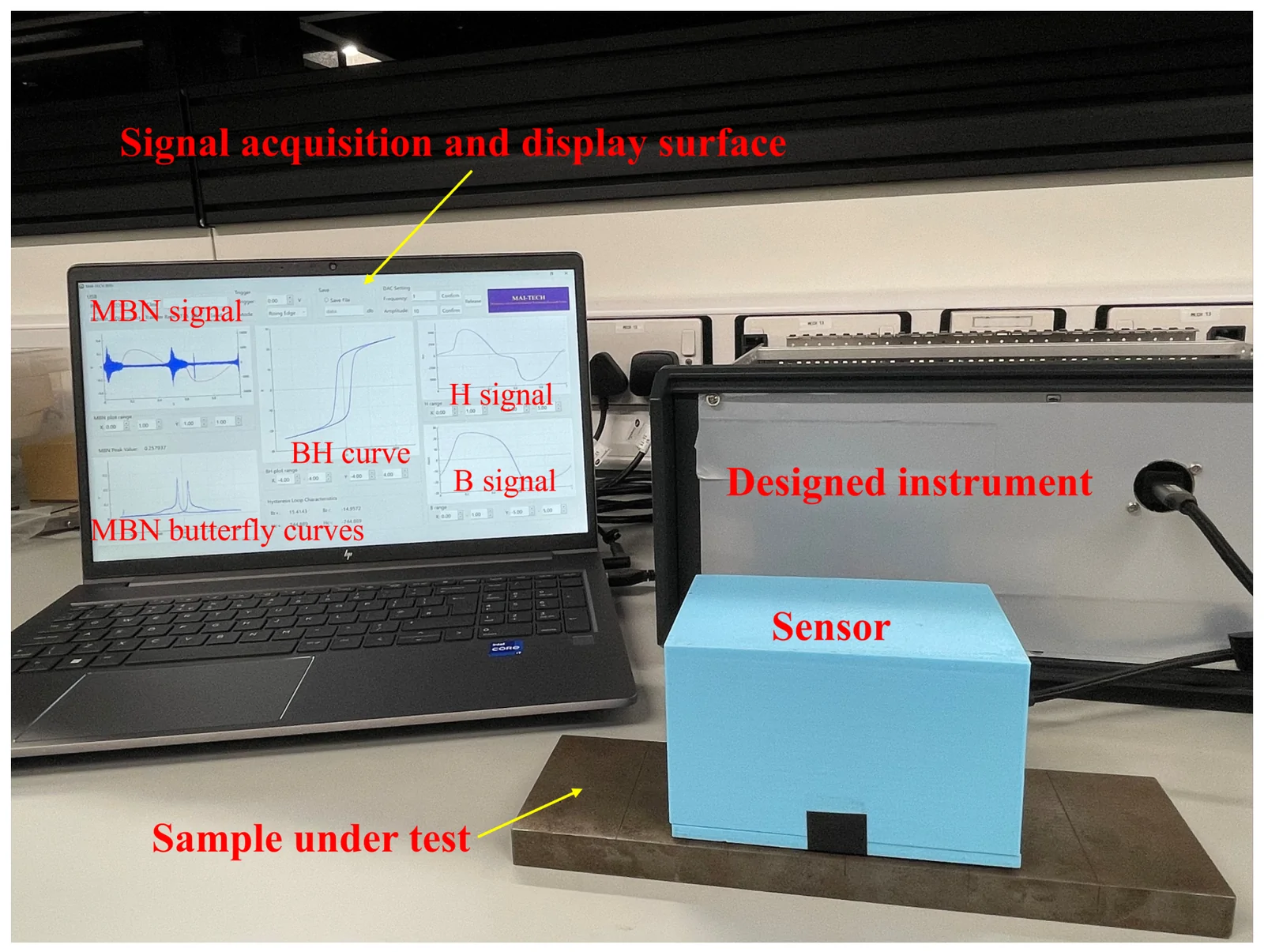
Experimental platform showing the instrument, probe, specimen and signal-acquisition interface.

**Figure 7:**
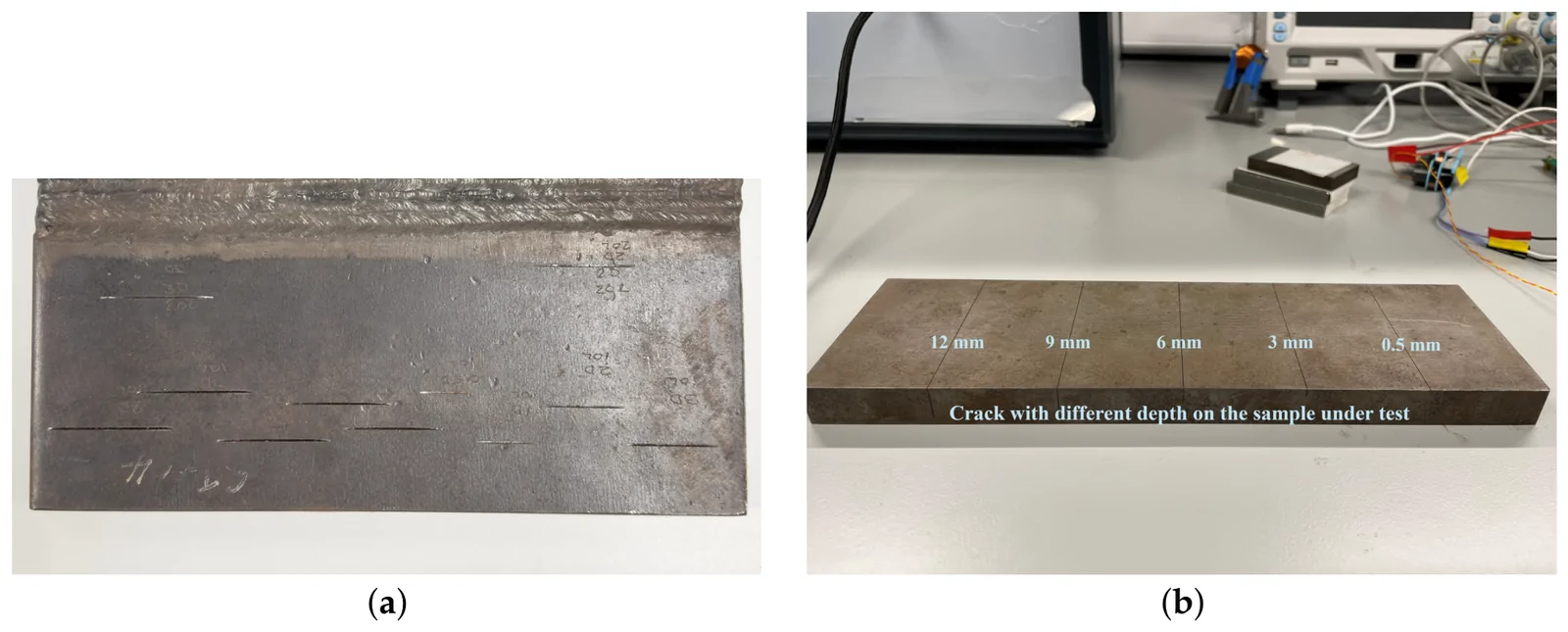
Steel test specimens containing (**a**) strip defects and (**b**) crack-like defects of different nominal depths.

The experimental platform comprised the custom instrument, a sensor probe and a computer interface (Figure 6). A ferrite element in the MBN pickup assembly provided magnetic coupling, and shielded cabling was used to reduce environmental electromagnetic interference. Figure 7 shows the two carbon-steel specimens. The crack-like defects had nominal depths of 0.5, 3, 6, 9 and 12 mm; the strip defects had nominal depths of 0.5, 1, 2, 3, 4, 5, 6 and 7 mm. Each series included a no-defect reference. Throughout this article, depth denotes the nominal geometrical defect depth, rather than the electromagnetic sensing depth. The excitation frequency was 1 Hz. Each acquisition lasted 10 s and contained ten magnetisation cycles. The MBN envelope was extracted separately for each cycle before the ten envelopes were averaged.

## 4. Results and Discussion

### 4.1. Depth-Dependent Magnetic-Field and MBN Responses

**Figure 8:**
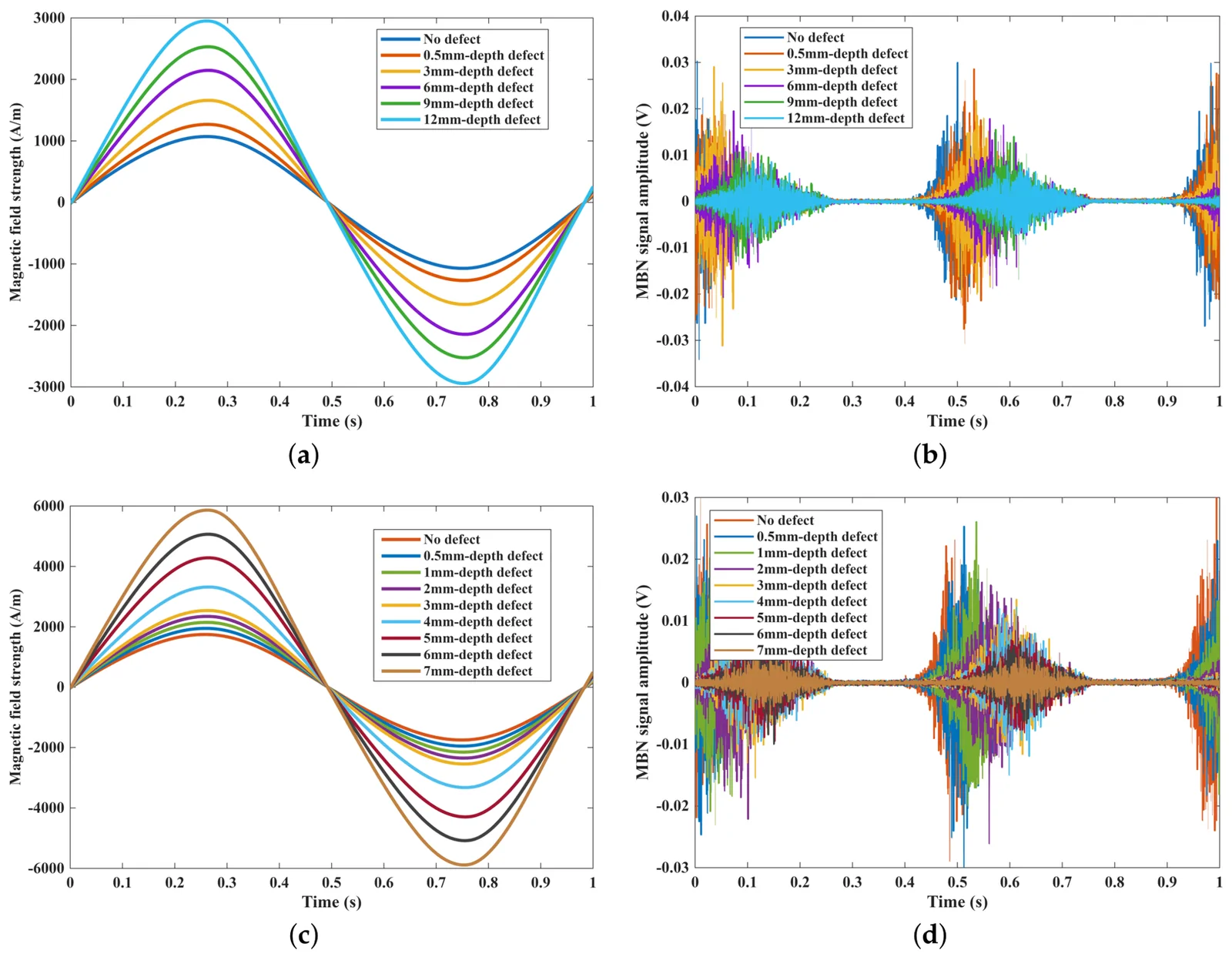
Measured signals for the no-defect references and different nominal defect depths: (**a**) magnetic field strength and (**b**) MBN voltage for crack-like defects; (**c**) magnetic field strength and (**d**) MBN voltage for strip defects.

The *H* and MBN signals were compared across the crack-like and strip defect series to examine how increasing depth affects the local magnetic field and the detected Barkhausen activity (Figure 8). The magnitude of *H* increases with defect depth, which is consistent with the simulated magnetic-circuit trend. The MBN burst amplitude generally decreases. These different responses are physically compatible: the Hall sensor measures the local magnetic field, whereas the pickup-coil voltage depends on the rate of Barkhausen-related flux change, as expressed by Equation (9). An increase in local *H* therefore does not imply an increase in MBN amplitude. The envelope analysis below examines this depth dependence over the magnetisation cycle.

### 4.2. Envelope Extraction and Feature Definition

The MBN envelope was estimated separately for each magnetisation cycle using square-law detection. The input voltage was squared without an additional gain factor, and the squared sequence was down-sampled and smoothed using a second-order Butterworth low-pass filter with unity gain at zero frequency. The smoothed mean-square estimate was then multiplied by two before taking its square root to obtain a sinusoidally calibrated root-mean-square (RMS) envelope. The processing sequence is defined by Equations (11)–(15):

(11) $$X\left(t\right)={\left[x(t)\right]}^{2}$$

(12) $$Y\left(t\right)=\mathrm{downsample}\left(X(t),N\right)$$

(13) $$\left[b,a\right]=\mathrm{butter}\left(n,w_{n}\right)$$

(14) $$H\left(z\right)=\frac{B(z)}{A(z)}=\frac{b_{1}z^{n}+b_{2}z^{n-1}+\cdots +b_{n+1}}{a_{1}z^{n}+a_{2}z^{n-1}+\cdots +a_{n+1}}$$

(15) $$Y_{e}\left(t\right)=\sqrt{2\;\mathrm{Filter}\left(b,a,Y(t)\right)}$$

where x(t) is the input MBN voltage, *N* is the down-sampling factor, and butter denotes the Butterworth-filter design operation. The filter order is n=2, and $w_{n}$ is the cutoff frequency normalised to the Nyquist frequency of the down-sampled sequence. The vectors *b* and *a* contain the numerator and denominator coefficients of H(z), and Filter denotes application of this digital low-pass filter. Both the squared signal and its smoothed mean-square estimate have units of $V^{2}$; the final envelope $Y_{e}\left(t\right)$ has units of V.

The amplitude normalisation follows from a locally sinusoidal signal $x\left(t\right)=A\left(t\right)\cos\left(\omega_{c}t+\phi \right)$, where A(t) varies slowly compared with the carrier period. In this case,

$$x^{2}\left(t\right)=\frac{A^{2}\left(t\right)}{2}\left[1+\cos(2\omega_{c}t+2\phi )\right].$$

When the low-pass filter preserves the slow variation and suppresses the component at twice the carrier frequency, its output is approximately $A^{2}\left(t\right)/2$. The single factor of two in Equation (15) therefore recovers A(t) after taking the square root. This is an amplitude calibration by a factor of $\sqrt{2}$ relative to the unscaled local RMS estimate; it is not a general correction for signal energy.

For the stochastic MBN signal, $Y_{e}\left(t\right)$ is interpreted as a sinusoidally calibrated local RMS envelope, rather than an exact instantaneous peak envelope. This interpretation assumes that the smoothing resolves the burst modulation and that aliasing introduced by down-sampling the squared sequence is negligible; low-pass filtering after down-sampling cannot remove components that have already aliased into the envelope band. The per-cycle envelopes were averaged before feature extraction. The RMS feature reported in Table 3 is the root-mean-square value of this averaged envelope over the analysis cycle and is distinct from the local RMS of the input voltage.

**Figure 9:**
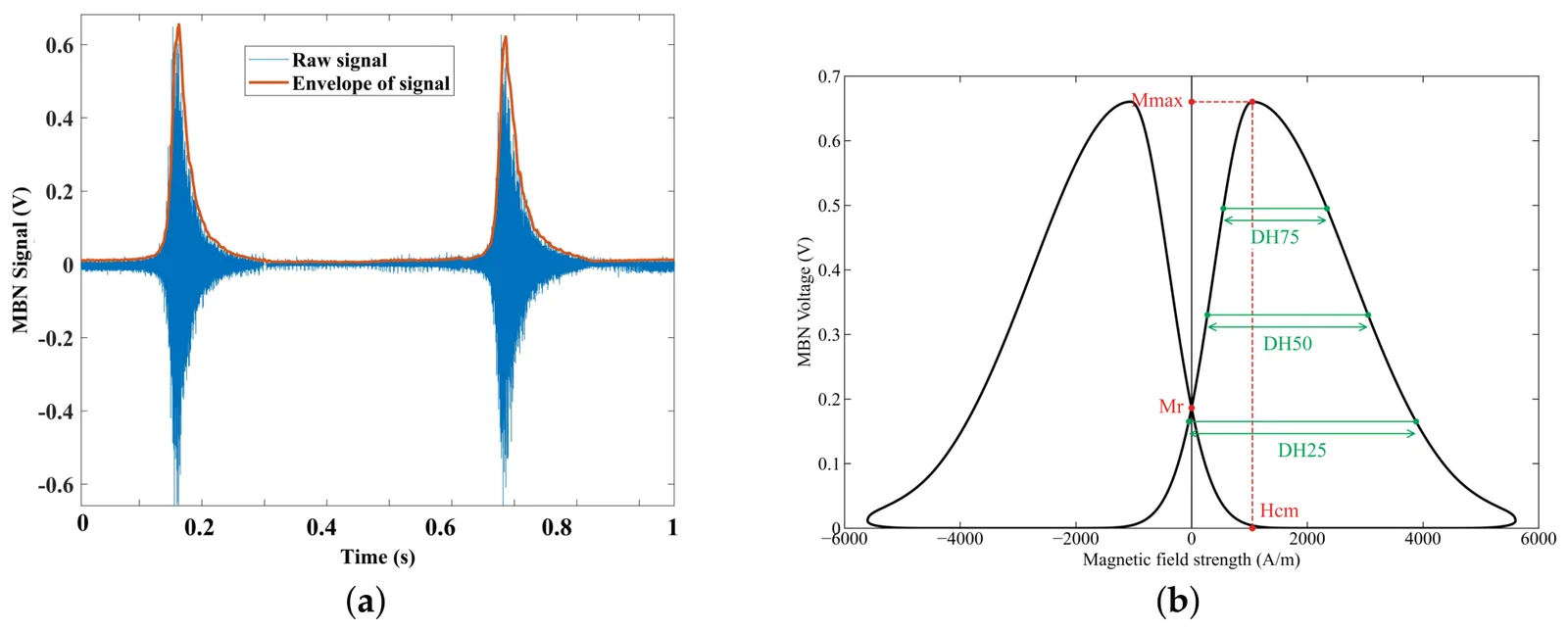
MBN signal processing and feature illustration: (**a**) example of a raw signal and its envelope, (**b**) schematic butterfly curve.

**Figure 10:**
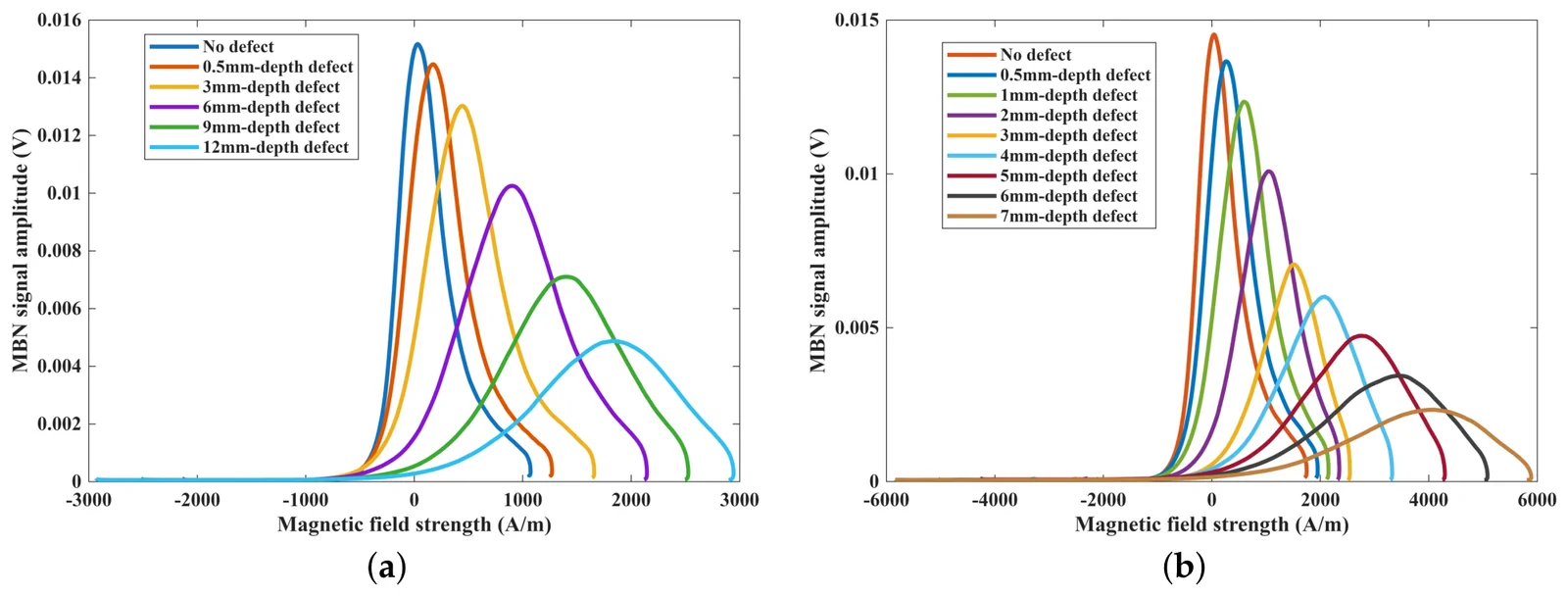
MBN envelope responses plotted against measured magnetic field strength for (**a**) crack-like defects and (**b**) strip defects, including the corresponding no-defect references.

Figure 9a illustrates the raw signal and its envelope. Pairing the averaged envelope with the measured *H* signal produces the field-dependent MBN response; Figure 9b schematically defines the associated amplitude, peak-field and width features. The experimental curves for the two defect series are shown in Figure 10, and the feature definitions are listed in Table 3. The voltage features $M_{\max}$, $M_{r}$ and $M_{\mathrm{mean}}$ describe the measured MBN response, rather than magnetisation. In particular, $M_{r}$ is the envelope amplitude at H=0, and $H_{cm}$ denotes the field at the envelope peak rather than a direct measurement of *B*–*H* coercivity.

### 4.3. Features Extracted from the MBN Butterfly Curves

**Figure 11:**
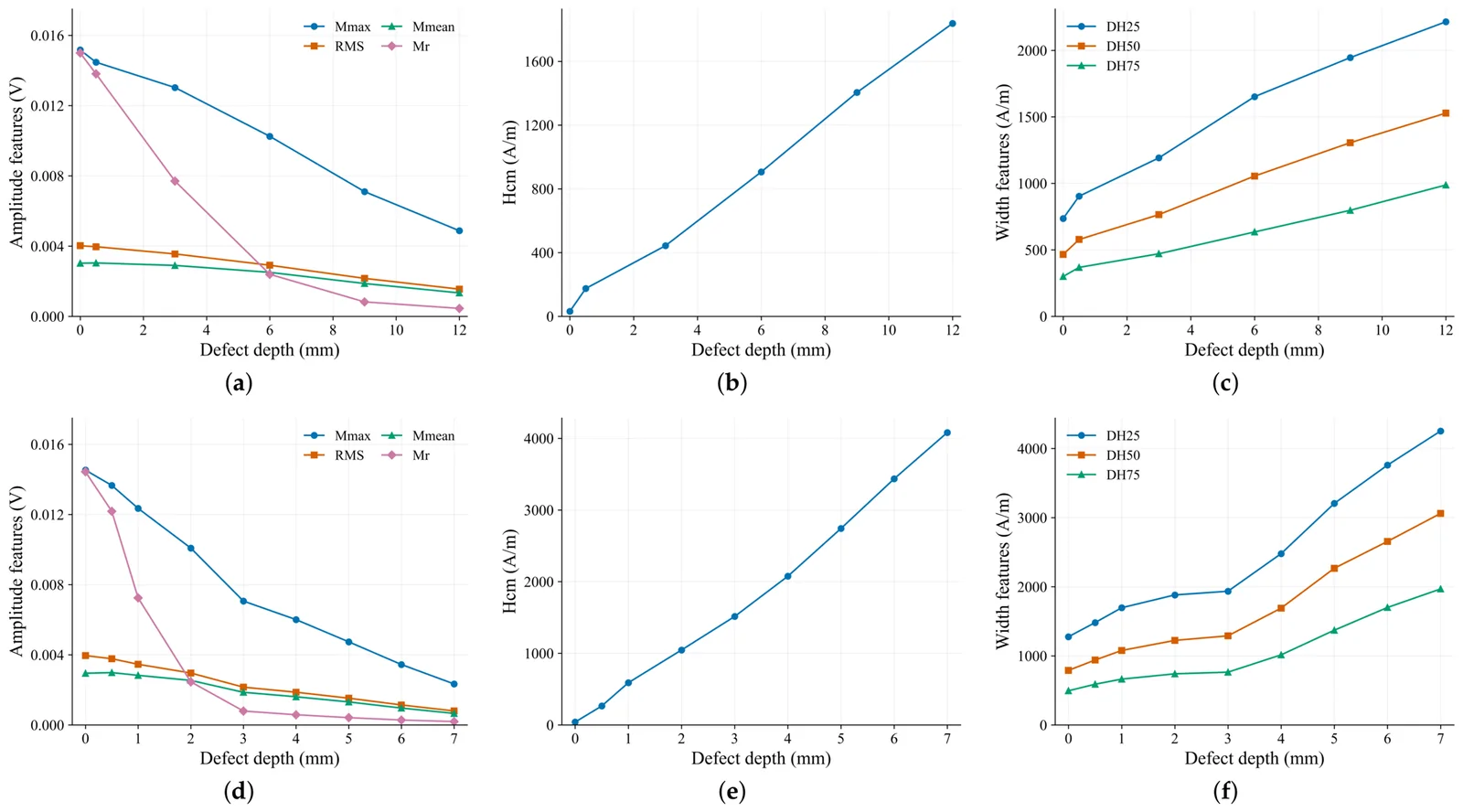
Dependence of MBN features on defect depth: (**a**–**c**) crack-like defects, (**d**–**f**) strip defects. (**a**,**d**) show amplitude features, (**b**,**e**) show $H_{cm}$, and (**c**,**f**) show width features.

Table 4 and Table 5 summarise the features extracted from the MBN butterfly curves, and Figure 11 shows their dependence on defect depth. Both defect series exhibit a reduction in amplitude accompanied by a shift of the envelope peak towards higher measured magnetic fields and an increase in envelope width. The no-defect reference for each series is plotted at 0 mm.

$M_{\max}$, RMS and $M_{r}$ decrease with increasing depth in both series, while $M_{\mathrm{mean}}$ decreases overall after a small initial increase relative to the no-defect reference. These features characterise the peak response, the root-mean-square envelope level, the response at zero measured field and the cycle-mean amplitude, respectively. Their combined behaviour shows that the depth dependence involves both the strongest MBN activity and the response over the magnetic cycle.

The amplitude trend is consistent with defect-induced changes in magnetisation and magnetic flux coupling. Equations (7) and (9) relate the detected voltage to irreversible magnetisation dynamics, the field sweep rate and the flux linked by the pickup coil [19,21]. A deeper discontinuity changes the magnetic reluctance and redistributes the field within the specimen–probe circuit. The simulated reduction in linked flux and increase in the Hall-sensor-domain response are qualitatively consistent with this interpretation. However, the macroscopic simulation alone does not establish the microscopic mechanism responsible for the measured MBN amplitude changes.

$H_{cm}$ increases with depth for both defect series, indicating that the maximum envelope response occurs at a higher measured field. Equation (7) relates Barkhausen activity to the irreversible differential susceptibility and the field sweep rate; the detected peak also depends on pickup-coil coupling and signal conditioning. The observed peak shift is consequently consistent with a change in the field-dependent magnetisation response as the defect modifies the local magnetic circuit. $H_{cm}$ complements the amplitude features by locating the strongest detected activity along the measured field axis.

$DH_{25}$, $DH_{50}$ and $DH_{75}$ also increase with depth in both series. As these widths are evaluated at 25%, 50% and 75% of the corresponding peak amplitude, their increase describes a broader response in magnetic-field space despite the reduction in peak height. The common trend at all three levels indicates broadening of both the central peak and its lower-amplitude flanks. In the theoretical framework above, this behaviour is consistent with a redistribution of the detected irreversible magnetisation activity over the measured field interval.

Together, the amplitude, peak-field and width features provide complementary indicators of defect depth: the envelope becomes weaker, shifts towards higher fields and broadens as depth increases. The agreement in trend between the two series supports their use for relative depth assessment. Their different magnitudes and rates of change also motivate calibration for the specific specimen and probe configuration.

Among the investigated features, the RMS of the averaged MBN envelope is recommended as a practical indicator for relative depth assessment because it decreases monotonically in both series and requires no mapping onto the H axis. For broader NDT applications, material and configuration specific calibration and combined use of amplitude, peak-field and width features should be considered and validated.

The proposed method focuses on depth assessment for defects with known geometry; defect localisation and shape recognition are outside the scope of this study. In practical applications, camera-based vision or another suitable method could first identify the defect’s location, shape and orientation to guide probe placement and alignment before MBN and H measurements.

## 5. Conclusions

Adding a dedicated MBN channel to the *B*–*H* measurement platform enabled defect-depth sensitivity to be examined together with the local magnetic-field response. Under the tested conditions, increasing defect depth was associated with a stronger measured *H* response alongside a weaker MBN response. The finite-element trends and the theoretical relation between irreversible magnetisation and induced pickup voltage suggest that field redistribution and magnetic coupling contribute to the observed depth sensitivity. Peak amplitude, RMS and zero-field amplitude decreased monotonically in both tabulated series, whereas the cycle-mean amplitude decreased overall after a small initial increase. Peak-field and width features increased with depth. These trends support relative depth assessment under the investigated conditions.

The present study proposes a combined H–MBN measurement approach and provides preliminary feature extraction and analysis for relative depth assessment under the investigated conditions. Because different probes were used for the two defect series, the effects of defect geometry and probe size remain coupled. Future work will compare both probes on common reference specimens, quantify repeatability and lift-off sensitivity, and evaluate calibrated H only, MBN only and combined depth-estimation models on independent specimens. These investigations will determine whether simultaneous measurements improve precision and establish the accuracy and operating range of quantitative defect sizing.

## Figures and Tables

**Table 1:** Material and geometric parameters of the finite-element model.

Component	Excitation Coils	Magnetic Induction Coil	Hall-Sensor Sampling Domain	Sample Under Test
Material	Copper	Copper	Air	Ferromagnetic plate
Dimensions (mm)	Wire diameter: 0.4 Height: 28	Wire diameter: 0.1 Height: 10	Length: 2 Width: 2 Height: 3	Length: 210 Width: 160 Height: 20
Number of turns	200	50	/	/
Relative permeability	0.99	0.99	1	100
Conductivity ($S\;m^{-1}$)	$5.71\times 10^{7}$	$5.71\times 10^{7}$	1	$2\times 10^{6}$

**Table 2:** Parameters of the MBN pickup coil.

Parameter	Value
Coil inner diameter (mm)	0.78
Coil outer diameter (mm)	2.23
Coil height (mm)	1.96
Number of turns	1000
Wire diameter (mm)	0.03
Inductance (μH)	619
Resistance (Ω)	125.5

**Table 3:** Definitions of features extracted from the MBN response.

Feature	Definition
$M_{\max}$ (V)	Maximum MBN envelope amplitude
$H_{cm}$ (A/m)	Measured magnetic field at the maximum envelope amplitude
$M_{r}$ (V)	MBN envelope amplitude at zero measured magnetic field (H=0)
RMS (V)	Square root of the mean squared amplitude of the averaged MBN envelope over the analysis cycle
$DH_{25}$ (A/m)	Magnetic-field width of the envelope peak at 25% of its maximum amplitude
$DH_{50}$ (A/m)	Magnetic-field width of the envelope peak at 50% of its maximum amplitude
$DH_{75}$ (A/m)	Magnetic-field width of the envelope peak at 75% of its maximum amplitude
$M_{\mathrm{mean}}$ (V)	Mean amplitude of the averaged MBN envelope over the analysis cycle

**Table 4:** Extracted MBN features for crack-like defects. Amplitude features are expressed in V; $H_{cm}$ and $DH_{25}$, $DH_{50}$ and $DH_{75}$ are expressed in $A\;m^{-1}$.

Feature/Defect Depth	0 mm	0.5 mm	3 mm	6 mm	9 mm	12 mm
$M_{r}$	0.0150	0.0138	0.0077	0.0024	0.0008	0.0005
$H_{cm}$	31.1761	174.7529	443.5016	905.7847	1404.5180	1837.8462
$M_{\max}$	0.01517	0.01447	0.01303	0.01025	0.00710	0.00488
RMS	0.00402	0.00396	0.00355	0.00291	0.00216	0.00155
$M_{\mathrm{mean}}$	0.00302	0.00304	0.00290	0.00251	0.00187	0.00133
$DH_{25}$	735.5154	903.5172	1191.6081	1651.7593	1945.5556	2215.3355
$DH_{50}$	465.4917	577.9507	764.8433	1054.9479	1305.7451	1528.3134
$DH_{75}$	299.7482	368.2002	470.5272	634.4694	797.5289	987.8988

**Table 5:** Extracted MBN features for strip defects. Amplitude features are expressed in V; $H_{cm}$ and $DH_{25}$, $DH_{50}$ and $DH_{75}$ are expressed in $A\;m^{-1}$.

Defect Depth	$M_{r}$	$H_{\mathrm{cm}}$	$M_{\max}$	RMS	$M_{\mathrm{mean}}$	${\mathrm{DH}}_{25}$	${\mathrm{DH}}_{50}$	${\mathrm{DH}}_{75}$
0 mm	0.0144	40.2676	0.0145	0.0040	0.0029	1276.3867	789.3796	494.1481
0.5 mm	0.0122	264.0600	0.0137	0.0038	0.0030	1480.4304	938.8572	588.9371
1 mm	0.0072	588.9161	0.0123	0.0035	0.0028	1695.7047	1077.4088	662.8519
2 mm	0.0025	1045.4305	0.0101	0.0030	0.0025	1881.1317	1222.8580	739.6352
3 mm	0.0008	1513.3738	0.0071	0.0022	0.0019	1934.8435	1289.2035	763.3782
4 mm	0.0006	2075.1804	0.0060	0.0019	0.0016	2478.8585	1690.2731	1013.9933
5 mm	0.0004	2741.6796	0.0047	0.0015	0.0013	3205.3108	2267.1851	1370.5076
6 mm	0.0003	3434.8912	0.0034	0.0011	0.0010	3759.4568	2655.5576	1699.3664
7 mm	0.0002	4081.3840	0.0023	0.0008	0.0007	4253.1776	3061.0828	1967.9248

## Data Availability

The data presented in this study are not publicly available.

## Abbreviations

The following abbreviations are used in this manuscript:

B–H	Magnetic flux density–magnetic field strength
J–A	Jiles–Atherton
MBN	Magnetic Barkhausen noise
NDT	Non-destructive testing
RMS	Root-mean-square
